# Clinical determinants and prognostic significance of hypocapnia in acute heart failure

**DOI:** 10.1038/s41598-022-20525-9

**Published:** 2022-10-07

**Authors:** Mateusz Garus, Agata Zdanowicz, Marat Fudim, Robert Zymliński, Piotr Niewiński, Bartłomiej Paleczny, Marta Rosiek-Biegus, Gracjan Iwanek, Piotr Ponikowski, Jan Biegus

**Affiliations:** 1 Institute of Heart Diseases, Medical University, ul. Borowska 213, 50-556 Wroclaw, Poland; 2 Department of Cardiology, Duke University School of Medicine, Durham, NC USA; 3 Department of Physiology and Pathophysiology, Medical University, Wroclaw, Poland; 4 Department of Internal Medicine, Pneumology and Allergology, Medical University, Wroclaw, Poland

**Keywords:** Cardiology, Heart failure

## Abstract

The aim of this research was to examine the prevalence of hyperventilation (defined by pCO_2_ value) among acute heart failure (AHF) patients and to link it with potential triggers and prognosis. All patients underwent dyspnea severity assessment and capillary blood examination on hospital admission and during hospitalization. Out of 241 AHF patients, 57(24%) were assigned to low pCO_2_ group (pCO_2_ ≤ 30 mmHg) and 184 (76%) to normal pCO_2_ group (pCO_2_ > 30 mmHg). Low pCO_2_ group had significantly lower HCO_3_^-^ (22.3 ± 3.4 vs 24.7 ± 2.9 mmol/L, p < 0.0001) and significantly higher lactate level (2.53 ± 1.6 vs 2.14 ± 0.97 mmol/L, p = 0.03). No differences between groups were observed in respect to the following potential triggers of hyperventilation: hypoxia (sO_2_ 92.5 ± 5.2 vs 92 ± 5.6% p = 0.57), infection (CRP 10.5[4.9–26.4]vs 7.15[3.45–17.35] mg/L, p = 0.47), dyspnea severity (7.8 ± 2.3vs 8.0 ± 2.3 points, p = 0.59) and pulmonary congestion (82.5 vs 89.1%, p = 0.19), respectively. Low pCO_2_ value was related to an increased 4-year all-cause mortality hazard ratio (HR) (95% CI) 2.2 (1.3–3.6); p = 0.002 and risk of death and of rehospitalization for HF, HR (95% CI) 2.0 (1.3–3.0); p = 0.002. Hyperventilation is relatively frequent in AHF and is related to poor prognosis. Low pCO_2_ was not contingent on expected potential triggers of dyspnea but rather on tissue hypoperfusion.

## Introduction

Dyspnea is a fundamental clinical sign in patients hospitalized for acute heart failure (AHF). There are several potential causes of dyspnea that may further lead to hyperventilation in AHF, like: congestion, including pulmonary congestion (promoting hypoxemia), infection, hypoperfusion, hypoxia, activation of hormonal axis (renin–angiotensin–aldosterone), iron deficiency as well as chemoreceptor overactivation. The imbalance in several cardiorespiratory reflex arcs that control and adjust adequate ventilatory and hemodynamic response to changing environmental conditions is a well-known element of heart failure (HF) pathophysiology^1–3^. The increased ventilatory response to hypoxia/hypercapnia mediated by oversensitivity of peripheral chemoreceptors is related to a worse clinical phenotype in chronic HF, including: low exercise tolerance, increased dyspnea sensation as well as high mortality^4–6^.

Given the fact, that the carbon dioxide (CO_2_) easily diffuses through the capillaries and is readily exchanged at the pulmonary alveolus level, it may serve as a valid indicator of the ventilatory effort with hypocapnia being a marker of hyperventilation.

The data quantifying ventilatory response and hypocapnia in AHF settings is limited. The prognostic significance of hypocapnia among AHF patients has been shown before. However, little is known regarding dynamics of changes in partial pressure of carbon dioxide (pCO_2_) during the hospital stay and clinical determinants of hyperventilation (and hypocapnia) in the settings of AHF.

## Materials and methods

### Study population

This is a single-centre, observational study. The study population included patients admitted with AHF to the Centre of Heart Diseases. 4th Military Hospital, Wroclaw, Poland. All participants were enrolled in the AHF registry carried out between January 2016 and September 2017.

The inclusion criteria were as follows: adult age (≥ 18 years old), AHF as a primary cause of hospitalization, administration of intravenous furosemide at admission and a written informed consent provided by the patient. Exclusion criteria were: cardiogenic shock, diagnosis of acute coronary syndrome, known severe liver disease, end-stage renal disease requiring renal replacement therapy.

AHF was defined according to the criteria of the European Society of Cardiology [ESC] guidelines.

Written informed consent was obtained from all patients. This study was approved by local ethics committee (Komisja Bioetyczna. Wroclaw Medical University) and performed in accordance with Declaration of Helsinki and Good Clinical Practice.

### Study design

Following admission to hospital clinical examination and detailed information related to subject’s demographics (including history of HF), comorbidities, previous treatment and findings obtained from physical examination were recorded. Assessment of dyspnea severity was performed with the use of a self-reported 10-point Likert scale (where 0 corresponds to “absence of dyspnea” and 10 corresponds to “dyspnea of the worst severity/maximal dyspnea”).

Venous blood collection, capillary blood gas analyses and assessment of the clinical status were carried out at baseline and at the following subsequent timepoints: day-1 and discharge. The samples were collected, centrifuged and frozen (at − 70 °C) for additional analysis.

### Laboratory measurements in peripheral blood

The following laboratory parameters were measured using standard methods in our laboratory:

- blood gas analysis was performed with the use of capillary blood from finger at a baseline and at the following subsequent timepoints: day-1 and discharge. A specimen was analysed with the use of automated blood gas analyser in the hospital’s laboratory (ABL FLEX, Radiometer).
- plasma NTproBNP (N-Terminal Pro-B-Type Natriuretic Peptide) (method: immunoenzymatic, Siemens, Marburg, Germany); plasma cardiac troponin (TNI) (method: immunoenzymatic, single Dimension RxLMax, Siemens)

The following parameters were measured from initially frozen samples:

- markers of inflammation: Interleukin (IL)-6 and interleukin (IL)-22 measured with the use of The Quantikine ELISA Immunoassay kit (R&D Systems. Inc., Minneapolis. MN. USA)
- serum sTfR (mg/L) immunonephelometric technique (Siemens Healthcare Diagnostics. Inc., Deerfield, IL, USA).
- RAAS (renin and aldosterone system) activation method: chemiluminescent immunoassay-CLIS, LIASON.

The chest X-rays results have been reviewed and radiological assessment of pulmonary congestion has been classified as follows: (1) no radiological sings of congestion; (2) radiological sings of congestion (pleural effusion, any signs of congestion not classified as radiological signs of pulmonary edema); (3) radiological signs of severe pulmonary congestion.

On the basis of a current literature review, potential triggers of hyperventilation were identified and investigated. The selected triggers comprised: anaemia (expressed by: haemoglobin, haematocrit). hypoxia (expressed by: partial pressure of O_2_ (pO_2_)_,_ oxygen saturation (sO_2_)), infection (represented by: white blood cell count, C-reactive protein), hypoperfusion (systolic blood pressure, pH, lactates), sensation of dyspnea at admission (measured by predefined scale), congestion (presence of pulmonary congestion at admission, NTproBNP), iron deficiency (expressed by ferritin, sfTR (solube transferrin receptor)) and RAAS activation (measured by renin and aldosterone serum concentration).

### Categorization

Value of pCO_2_ was used as a representation of ventilatory status of the patient. The pCO_2_ cut-off on admission was set up at 30 mmHg, upon this value patients were divided to either low pCO_2_ group (pCO_2_ ≤ 30 mmHg) or to normal pCO_2_ group (pCO_2_ > 30 mmHg). The cut-off value used to identify low pCO_2_ group was established arbitrary with reference to the literature^7–9^.

The following signs of HF were examined: (1) oedema (with the use of 0–3-point scale, where 3 corresponds to most severe oedema). (2) pulmonary congestion (with the use of 0–3-point scale, where 3 corresponds to congestion reaching upper parts of lungs) and (3) jugular venous pressure (JVP).

### Study outcomes

The clinical endpoints of the study were:

1. In-hospital mortality;
2. One-year all-cause mortality;
3. Composite endpoint of 1-year all-cause mortality and rehospitalization for the HF.

### Clinical follow-up

Discharged patients were monitored according to the HF clinic surveillance protocols for at least 1 year. Several sources were used to collect subsequent data regarding patients rehospitalization and survival status, including: patients' testimony, relatives (phone-based interviews), relevant clinic database and/or national register of citizens. Not a single patient was lost to follow-up.

### Statistical analysis

Continuous variables with a normal distribution were presented as mean ± standard deviation, variables with skewed distribution were described by medians with [upper and lower quartiles], categorized variables were indicated as numbers and percentages. Statistical analysis between study groups were conducted with the use of T-test, Mann–Whitney U-test or χ^2^. The Cox proportional hazards models were utilized to calculate the hazard ratio (HR) with corresponding 95% confidence interval (95% CI) for all-cause mortality. Multivariable analysis was adjusted for confounding variables: age, left ventricular ejection fraction (LVEF), systolic blood pressure at admission, hemoglobin, NTproBNP and blood urea nitrogen. Kaplan–Meier survival curves were used for visualization of survival analysis.

The p value of < 0.05 was considered to be statistically significant. STATISTICA 13 (StatSoft) was utilized to perform statistical analysis.

## Results

### Baseline characteristics

The study population included 241 AHF patients. predominantly male (72%), with a mean age of 70 ± 13 years. The mean systolic/diastolic blood pressure and heart rate at admission were: 134 ± 32/79 ± 16 mmHg and 90 ± 24 beats per minute, respectively. The mean LVEF was: 39.5 ± 15% with a predominant ischemic etiology (n = 122, 51%). The median of NTproBNP and plasma Troponin I on admission were 5.659 [3.368–11.920] pg/mL and 0.06 [0.03–0.16] ng/mL, respectively. The mean degree of dyspnea reported by the patient on admission was 7.9 ± 2.3 points.

The mean: pH, pO_2_, pCO_2_, HCO_3_^-^ were 7.43 ± 0.07, 69.33 ± 19.86 mmHg, 35.17 ± 6.68 mmHg and 23.04 ± 3.61 mmol/L, respectively. Table 1 represents detailed information regarding patients’ baseline characteristics.

**Table 1:** Baseline characteristics.

Parameter	All patients
Number of patients	241 (100%)
Gender (male)	174 (72%)
Age (years)	70 ± 12.7
Heart rate (beat/min)	90 ± 24
Systolic blood pressure (mmHg)	134 ± 32
Diastolic blood pressure (mmHg)	79 ± 16
Left ventricle ejection fraction (%)	39.5 ± 14.9
Acute heart failure *(*de novo*)*	97 (40%)
**Heart failure etiology**	
Ischemic	122 (51%)
**Clinical assessment of pulmonary congestion**	
No congestion	30 (12.4%)
< 1/3 pulmonary area	131 (54.4%)
1/3–2/3 pulmonary area	58 (24.1%)
> 2/3 pulmonary area	22 (9.1%)
Radiological assessment of pulmonary congestion*	
No radiological sings of congestion	51 (22%)
Radiological sings of congestion**	173 (73%)
Radiological signs of severe pulmonary congestion	37 (16%)
**Ventilation support**	
Non-invasive ventilation (yes)	17 (7,1%)
Intubation (yes)	4 (1,66%)
Pleural paracentesis during hospitalization (yes)	13 (5,4%)
Patient’s reported dyspnea on admission (points)	7.98 ± 2.31
**Blood count**	
Haemoglobin (g/dL)	13.3 ± 2.0
Haematocrit (%)	40 ± 5.6
White blood cells (g/L)	9.2 ± 4.4
Platelets (g/L)	208.7 ± 85.9
**Components of gas blood test**	
pH (potential hydrogen)	7.43 ± 0.07
pCO_2_ (partial pressure of carbon dioxide) (mmHg)	35.17 ± 6.68
pO_2_ (partial pressure of oxygen) (mmHg)	69.33 ± 19.86
SO_2_ (oxygen saturation) (%)	92.11 ± 5.47
HCO_3_^-^ (bicarbonates) (mmol/L)	23.04 ± 3.61
Lactates (mmol/L)	2.23 ± 1.16
C-reactive protein (mg/L)	7.7 [4.2–18.9]
Serum Na^+^ (mmol/L)	139 ± 4.4
Serum K^+^ (mmol/L)	4.23 ± 0.65
Creatinine (mg/dL)	1.36 ± 0.52
Urea (mg/dL)	59.5 ± 31.47
NTproBNP (pg/mL)	5659 [3368 – 11920]
Troponin I (ng/mL)	0.06 [0.03–0.16]
Total bilirubin (mg/dL)	1.03 [0.72–1.71]
Direct bilirubin (mg/dL)	0.5 [0.37–0.77]
Alanine transaminase (U/L)	31 [21 – 56]
Aspartate transaminase (U/L)	28 [22 – 41]
Albumin (g/dL)	3.78 ± 0.4

*Data available in n = 237.

**Defined as pleural effusion, any signs of congestion not classified as radiological signs of pulmonary edema.

### Prevalence of hypocapnia during hospitalization for AHF

**Figure 1:**
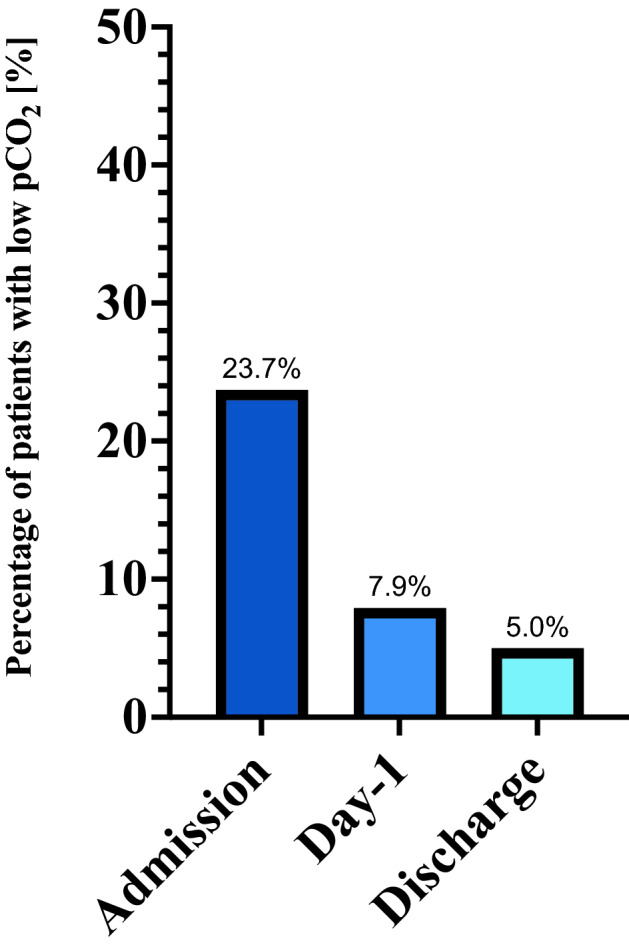
The prevalence of low pCO_2_ (≤ 30 mmHg) in AHF patients during hospital stay.

There were 57 (24%) patients classified as low pCO_2_ and 184 (76%) classified as normal pCO_2_. The percentage of patients with low pCO_2_ was 23.7% on admission 7.9% at day-1 and 5.0% at discharge (Fig. 1).

### Comparison of patients with low vs normal pCO_2_ on admission

The low pCO_2_ group had significantly lower systolic blood pressure (123 ± 27 vs 137 ± 32 mmHg, p = 0.004) significantly higher lactate (2.53 ± 1.6 vs 2.14 ± 0.97 mmol/L, p = 0.03) and lower HCO_3_^-^ (22.3 ± 3.4 vs 24.9 ± 2.9 mmol/L, p < 0.0001) at baseline. Moreover, the low pCO_2_ group had significantly higher levels of NTproBNP 7.493 [5016–16395] vs 5.202 [3068–10152] pg/mL, p = 0.004.

There were no differences between the studied groups in respect to the following potential triggers of hyperventilation: anaemia (e.g., haemoglobin 13.36 ± 2 vs 13.25 ± 2 mg/dL, p = 0.72), hypoxemia (sO_2_ 92.5 ± 5.2 vs 92 ± 5.6%, p = 0.57), infection status (e.g., CRP 10.5 [4.9–26.4] vs 7.15 [3.45–17.35] mg/L, p = 0.47; IL-6 9.7 [0.5–20.9] vs 8.3 [1.0–21.4] pg/mL, p = 0.93;

IL-22 7.0 [2.0–25.0] vs 6.5 [0.0–18.5] pg/mL, p = 0.18); severity of dyspnea (7.8 ± 2.3 vs 8.0 ± 2.3 points, p = 0.59) and presence of pulmonary congestion (82 vs 89.1%, p = 0.19), respectively. Moreover, there were no differences in RAAS activity and iron status between the groups. The differences between selected, potential determinants of hyperventilation are shown in Table 2.

**Table 2:** Comparison of selected, potential triggers of hyperventilation by pCO_2_ level on admission.

Parameter	pCO_2_ partial pressure group		p
	≤ 30 mmHg n = 57 (24%)	> 30 mmHg n = 184 (76%)	
History of pulmonary disease	5 (8.8%)	22 (12%)	0.49
History of thyroid disease	9 (16%)	26 (14%)	0.75
**Anaemia**			
Haemoglobin (g/dL)	13.36 ± 2	13.25 ± 2	0.72
Haematocrit (%)	40 ± 5.8	40.1 ± 5.5	0.81
**Hypoxemia**			
pO_2_ (mmHg)	69.2 ± 13.1	69.4 ± 21.7	0.94
sO_2_ (%)	92.5 ± 5.2	92 ± 5.6	0.57
**Infection**			
White blood cells (G/L)	9.5 ± 5.1	9.0 ± 4.2	0.49
C-reactive protein (mg/L)	10.5 [4.9–26.4]	7.15 [3.45–17.35]	0.47
IL-6 (pg/mL)	9.7 [0.5–20.9]	8.3 [1.0–21.4]	0.93
IL-22 (pg/mL)	7.0 [2.0–25.0]	6.5 [0.0–18.5]	0.18
**Hypoperfusion/hypoxia**			
Systolic blood pressure (mmHg)	123 ± 27	137 ± 32	0.004
pH	7.46 ± 0.06	7.43 ± 0.07	0.005
HCO_3_^-^ (mmol/L)	22.3 ± 3.4	24.7 ± 2.9	< 0.0001
Lactates (mmol/L)	2.53 ± 1.6	2.14 ± 0.97	0.03
**Dyspnea at admission**			
Dyspnea at admission (points)	7.8 ± 2.3	8.0 ± 2.3	0.59
**Congestion**			
Clinical assessment of pulmonary congestion at admission (yes)	47 (82.5%)	164 (89.1%)	0.19
Radiological assessment of pulmonary congestion*			
No radiological sings of congestion	11 (20%)	40 (22%)	0.75
Radiological sings of congestion**	42 (76%)	131 (72%)	0.52
Radiological signs of severe pulmonary congestion	5 (9%)	32 (17%)	0.12
NTproBNP (pg/mL)	7492.5 [5015.5–16,394.5]	5201.5 [3068–10152]	0.004
**Ventilation support**			
Non-invasive ventilation (yes)	1 (2%)	16 (9%)	0.07
Intubation (yes)	1 (2%)	3 (2%)	0.94
Pleural paracentesis during hospitalization (yes)	2 (4%)	11 (6%)	0.49
**Iron status**			
Fe (g/dL)	55.7 ± 27.3	56.4 ± 30.5	0.89
Total iron binding capacity (g/dL)	349.6 ± 58	346.2 ± 73.1	0.76
sTfR at admission (mg/L)	2.2 ± 0.7	1.9 ± 0.9	0.07
Ferritin (g/L)	105.0 [81.0–219.0]	154.5 [83.5–247.5]	0.16
**RAAS activation**			
Renin (μIU/mL)	33.5 [4.2–262.3]	27.7 [6.8–96.6]	0.52
Aldosterone (ng/dL)	12.5 [6.9–29.6]	10.5 [6.5–15.7]	0.17
**Organ dysfunction**			
Serum Na^+^ (mmol/L)	136.5 ± 5.15	139.7 ± 3.8	0.0
Serum K^+^ (mmol/L)	4.34 ± 0.7	4.2 ± 0.6	0.13
Creatinine (mg/dL)	1.48 ± 0.6	1.32 ± 0.5	0.04
Spot urine sodium at admission (mmol/L)	80 ± 35	89 ± 35	0.13
Spot urine sodium at day-1 (mmol/L)	74 ± 34	76 ± 40	0.66
Urea (mg/dL)	68.7 ± 39	56.7 ± 28	0.02
Troponin I (ng/mL)	0.06 [0.03–0.11]	0.06 [0.03–0.2]	0.6
Total bilirubin (mg/dL)	1.6 [0.9–2.2]	0.9 [0.7–1.5]	0.02
Direct bilirubin (mg/dL)	0.7 [0.5–1]	0.48 [0.34–0.7]	0.03
Alanine transaminase (U/L)	37 [22–70.5]	30 [21–50]	0.01
Aspartate transaminase (U/L)	30.5 [24.5–64]	28 [20–40]	0.02
Albumin (g/dL)	3.72 ± 0.34	3.8 ± 0.4	0.22

*Data available in n = 237.

**Defined as pleural effusion, any signs of congestion not classified as radiological signs of pulmonary edema.

### Comparison of patients with low vs normal pCO_2_ at discharge

Patients with low pCO_2_ (< 30 mmHg) at discharge had significantly lower HCO_3_^−^ (20.6 ± 1.7 vs 23.3 ± 3.5 mmol/L, p < 0.05) and higher NTproBNP (6953 [2977–11859] vs 3029 [1830–6282] pg/mL, p < 0.05), while there was no difference in systolic blood pressure when compared to the rest of the population. There was a significant correlation between discharge pCO_2_ and: discharge NTproBNP R-Spearman correlation coefficient: − 0.15 and discharge lactate: − 0.14, both p < 0.05.

### Dyspnea and its change during hospitalization

**Figure 2:**
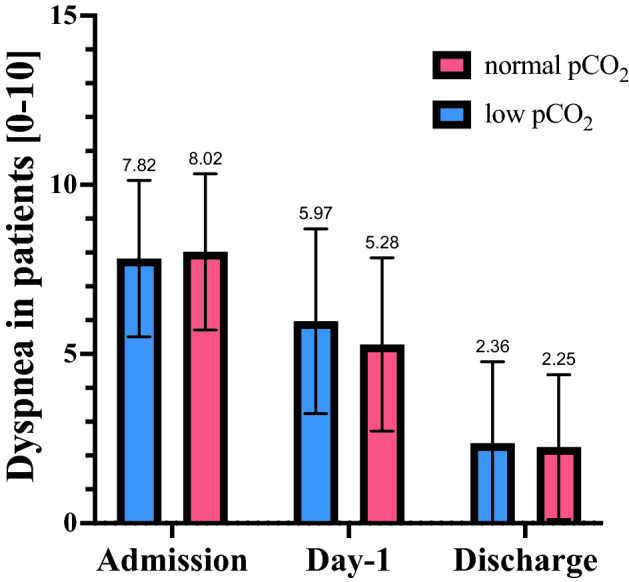
Comparison of dyspnea perception between patient with different pCO_2_ level. Assessment of dyspnea perception was performed with the use of a self-reported 10-point Likert scale. Red—patients with normal pCO_2_ (> 30 mmHg). Blue—patients with low pCO_2_ (≤ 30 mmHg).

Patients with low vs normal pCO_2_ reported comparable level of dyspnea sensation on admission (7.83 ± 2.3 vs 8.03 ± 2.3 points), day-1 (5.98 ± 2.3 vs 5.28 ± 2.3 points) and at discharge (2.86 ± 2.3 vs 2.24 ± 2.3 points); all p > 0.05. Patients from both groups experienced an improvement at day-1 (vs admission) (p < 0.05). As presented in Fig. 2 the improvement in group A and group B was of similar magnitude (all p > 0.05).

### Clinical and laboratory associations of hyperventilation on admission

The correlates of pCO_2_ are presented in Table 3. The pCO_2_ was independently associated with renin, oxygen saturation and HCO_3_^-^, with standardized regression coefficients: 0.15, 2.63, -2.22, respectively, all p < 0.05.

**Table 3:** Clinical and laboratory determinants of hyperventilation (pCO_2_ ≤ 30 mmHg on admission).

Variable	Univariable		Multivariable (intercept = 0)	
	Pearson correlation coefficient	p	Standardized regression coefficient*	p
sfTR (mg/L)	− 0.18	0.02		
Renin (μIU/mL)	− 0.18	0.01	0.15	< 0.05
Hemoglobin (g/dL)	− 0.05	0.53		
pO_2_ (mmHg)	0.02	0.76		
sO_2_ (%)	− 0.22	< 0.01	2.63	< 0.01
CRP (mg/L)	− 0.04	0.57		
HCO_3_^-^ (mmol/L)	0.14	0.06	− 2.22	< 0.01
Lactate (mmol/L)	0.04	0.58		
Dyspnea (points)	0.14	0.06		
Systolic blood pressure (mmHg)	0.35	0.00		
NT-proBNP at admission (pg/mL)	− 0.18	0.02		
Aldosterone (ng/dL)	− 0.29	< 0.01		

*sFTR* soluble transferrin receptor, *pO*_*2*_ partial pressure of oxygen, *sO*_*2*_ oxygen saturation, *CRP* C-reactive protein, *HCO*_*3*_^–^ bicarbonate, *NT-proBNP* N-terminal prohormone of brain natriuretic peptide.

*Progressive stepwise regression model – only final statistically significant variables of the model are presented.

### Prognostic significance of hyperventilation on admission

The in-hospital mortality was 4.1% (10 events), with a 1-year mortality of 29% (69 events). There was significantly higher proportion of patients who died during hospitalization in the low pCO_2_ group 8 (14%) when compared to the rest of the population 2 (1%). p < 0.0001. The pCO_2_ expressed as a continuous variable did not impact the 1-year outcomes. However, the low pCO_2_ group had significantly worse outcomes even after adjustments for well-defined prognosticators.

**Figure 3:**
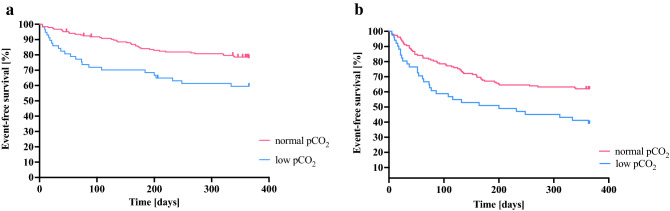
Kaplan–Maier curves for death or heart failure rehospitalization (whichever occurred first) by pCO_2_ level on admission. (**a**) Death analysis. Log rank p = 0.002. Red line—patients with normal pCO_2_ value (> 30 mmHg). Blue line—patients with low pCO_2_ value (≤ 30 mmHg). (**b**) Death or heart failure rehospitalization (whichever occurred first) by pCO_2_ level on admission. Log-rank p = 0.002. Red line—patients with normal pCO_2_ value (> 30 mmHg). Blue line—patients with low pCO_2_ value (≤ 30 mmHg).

Low pCO_2_ group as shown in Table 4 was associated with an increased 1-year all-cause mortality hazard ratio (HR) (95% CI) 2.2 (1.3–3.6); p = 0.002 and an increased risk of death and rehospitalization for HF (HR) (95% CI) 2.0 (1.3–3.0); p = 0.002. The Kaplan–Meier curves present the differences in mortality, based on pCO_2_ level groups (Fig. 3a,b).

**Table 4:** One-year all-cause mortality and heart failure rehospitalizations risks in relation to pCO_2_ level on admission.

	HR (95% CI) Univariate model	HR (95% CI) Multivariable model*
**360-day all-cause morality**		
pCO_2_ (mmHg)	0.97 (0.9–1.0); p = 0.33	
Low pCO_2_ group	2.2 (1.3–3.6); p = 0.002	1.8 (1.1–3.0) p < 0.01
**360-day all-cause mortality or rehospitalization for heart failure**		
pCO_2_ (mmHg)	0.97 (0.9–1.0); p = 0.25	
Low pCO_2_ group	2.0 (1.3–3.0); p = 0.002	1.6 (1.04–2.59); p < 0.05

*Adjusted for: age, ejection fraction, systolic blood pressure at admission, haemoglobin. NTproBNP and serum creatinine.

## Discussion

The broad implication of our study is that patients with heart failure and comorbid hypocapnia have worse long-term prognosis and poor clinical outcome.

Importantly, we have shown that low pCO_2_ was not associated with conventional clinical and laboratory triggers. The results presented here suggest that hypoperfusion was a key factor in the development of hypocapnia.

Dyspnea is the most common symptom among patients with HF and tends to be inevitably more severe as the disease progresses. The term itself refers to the subjective experience of breathing discomfort and is frequently described by patients as an inability to take a deep breath or a chest tightness. This symptom is complex in its etiology as there is a wide spectrum of pathophysiological triggers and mechanisms (cardiac/metabolic/neurogenic/pulmonary/haematological) involved in its development. Even though this phenomenon is widely studied, the diagnostic accuracy of dyspnea assessment seems to be limited due to lack of objective measures. The dyspnea in AHF might be related to hyperventilation (expressed by an increase in tidal volume and/or respiratory rate), but it may also be dependent on the subjective perception of unsatisfied inspiration caused by respiratory muscle weakness or dynamic lung hyperinflation^10^. Clinicians seldom precisely assess the specific physiological variables like respiratory rate, tidal volume or the minute ventilation in AHF patients. Therefore, there is a persistent unmet need for an objective identification of the patient’s ventilatory status. A growing body of evidence suggests that hyperventilation may contribute to the development of physiological derangements that result in HF progression^11–14^.

Here, we used pCO_2_ as surrogate of hyperventilation. We believe that the results of this study provide an insight into heart failure pathophysiology, relations of low pCO_2_ with potential clinical triggers of hyperventilation and prognostic significance of hypocapnia in AHF.

First, the results of our study reveal that there were discrepancies between pCO_2_ level/hyperventilation and patient self-reported dyspnea. The level of pCO_2_ did not affect the sensation of dyspnea reported by AHF patients. Therefore, it can be assumed that subjective dyspnea measures have inadequate diagnostic accuracy for identification of patients with disturbed breathing patterns and pCO_2_ seems to be more reliable indicator for detecting hyperventilation.

Second, hyperventilation was not contingent on evident and expected potential triggers such as anaemia (expressed by haemoglobin, haematocrit), infection (expressed by WBC, CRP, IL-6, IL-22), hypoxemia (expressed by pO_2_ and sO_2_) or pulmonary congestion on physical examination. However, hyperventilating patients had some markers of more advanced disease phenotypes and more severe multi-organ dysfunction. On the other hand, alternative markers of disease progression such as RAAS, iron status or spot urine sodium were comparable between the two groups.

Both groups differed in relation to markers of hypoperfusion and hypoxemia/hypoxia. A higher lactate level on admission was observed in the group with low pCO_2_. Lactate as a product of an anaerobic metabolism corresponds to impaired tissue perfusion. In response to energetic stress (as during AHF episode) energy demand increases and the sympathetic nervous system hyperactivates, all of which result in an increased glycolysis and lactate accumulation. Under conditions of insufficient tissue perfusion, persistent energy debt exceeds the buffering capacity of the body what consecutively leads to the development of hyperlacticaemia and metabolic acidosis. This goes also with agreement with the occurrence of metabolic acidosis (the lower bicarbonate levels) observed in the hyperventilating group. The metabolic acidosis may also be a surrogate of inadequate peripheral perfusion in AHF. As a result of bicarbonate depletion, ventilatory compensation (in the form of hyperventilation) emerges to maintain acid–base balance.

Therefore, it can be inferred that one of the mechanisms underlying hypocapnia in AHF is related to response to peripheral hypoperfusion. Thus, we may speculate that hypoperfusion (rather than direct hypoxemia) contributed to hyperventilation in an attempt to buffer the developing acidosis at the expense of CO_2_ loss. On the other hand, renin was independently related to hyperventilation, which may indicate that patients with more advanced stages of the disease (defined by elevated renin) as well as those with more severe metabolic collapse on admission to the hospital are more likely to develop hyperventilation.

As elegantly shown by Torres-Torrelo et al. peripheral chemoreceptors are also lactate sensors^15^. Thus, overactivation of the chemoreflex arc may be seen as hypothetical link between diminished tissue perfusion and increased ventilatory effort resulting in hypocapnia. It is also worth noting that HF individuals with hypersensitive peripheral chemoreceptors (~ 30% of HF patients) are characterized by worse prognosis. Therefore, it may be speculated that poor outcomes seen in our study in low pCO_2_ group might have been partly related to that fact. Assessment of peripheral chemosensitivity would definitely shed more light on the matter.

The association between hemodynamic impairment, enhanced sensitivity to carbon dioxide and Cheyne–Stokes respiration (CSR) among patients with HF is an additional contributing factor that should be taken under consideration^16^. Hyperventilation induced hypocapnia contributes to the ventilatory instability and the development of a periodic breathing with central sleep apnoea (CSA)^16^. Indeed, as it was presented by Naughton et al., patients with CSR-CSA had significantly lower values of pCO_2_ in comparison to patients who did not present this breathing pattern^17^.

It should be noted that, Cheyne-Stokes ventilation is a marker of a poor prognosis in HF and the central sleep apnoea is related to higher mortality risk in HF population^18^. Consequently, it can be assumed, that this aspect could have partially contributed to the poor clinical outcome observed in a group with hypocapnia. This assumption should be addressed in future studies.

Interestingly, despite clinical improvement 5% of patients had persistent hypocapnia at discharge. We may only speculate that low discharge pCO_2_ identifies patients with more profound metabolic misbalance/hypoperfusion that may be imperceptible from clinical perspective, analogically to those patients being discharged with residual congestion^19,20^. This theory may be supported by the fact that discharge pCO_2_ correlated with both discharge lactate and NTproBNP. However, we do not have a conclusive evidence that those two populations overlap in our study.

### Study limitations

This is a single-centre, observational study, which recruited relatively low number of patients. It should be emphasized that hypocapnia on admission for AHF might not be a simple function of hyperventilation. It is well possible that in some patients it was present even before the acute event. This could potentially be related to chronic renal disease with concomitant metabolic acidosis, the presence of periodic breathing or other unidentified factors—which by themselves may affect the survival. The pCO_2_ cut-off value used in our study was set up arbitrary (however in line with literature data), which is also an obvious limitation.

In conclusion, hypocapnia is relatively frequent among patients admitted to the hospital with AHF and related with poor prognosis. Low pCO_2_ was not contingent on evident and expected potential clinical and laboratory triggers, while tissue hypoperfusion seemed to play an important role in its’ development.

## Data Availability

The datasets used in the current research are available from the corresponding authors on reasonable request.

## References

[1] Ponikowski P (1997). Augmented peripheral chemosensitivity as a potential input to baroreflex impairment and autonomic imbalance in chronic heart failure. Circulation.

[2] Haack KKV, Marcus NJ, del Rio R, Zucker IH, Schultz HD (2014). Simvastatin treatment attenuates increased respiratory variability and apnea/hypopnea index in rats with chronic heart failure. Hypertension (Dallas, Tex.: 1979).

[3] Schultz HD, Marcus NJ, del Rio R (2015). Mechanisms of carotid body chemoreflex dysfunction during heart failure. Exp. Physiol..

[4] Ponikowski P, Banasiak W (2001). Chemosensitivity in chronic heart failure. Heart Fail. Monit..

[5] Toledo C (2017). Contribution of peripheral and central chemoreceptors to sympatho-excitation in heart failure. J. Physiol..

[6] Blain GM, Smith CA, Henderson KS, Dempsey JA (2010). Peripheral chemoreceptors determine the respiratory sensitivity of central chemoreceptors to CO(2). J. Physiol..

[7] Hayakawa J, Yoshida G, Usuda Y (1992). Effect of hypocapnia on arterial oxygenation during induced hypotension. Masui Jpn. J. Anesthesiol..

[8] Plöchl W (2001). Can hypocapnia reduce cerebral embolization during cardiopulmonary bypass?. Ann. Thorac. Surg..

[9] Kato T (2021). Prognostic effects of arterial carbon dioxide levels in patients hospitalized into the cardiac intensive care unit for acute heart failure. Eur. Heart J. Acute Cardiovasc. Care.

[10] O’Donnell DE, Laveneziana P (2006). Physiology and consequences of lung hyperinflation in COPD. Eur. Respir. Rev..

[11] Hawkins SM, Guensch DP, Friedrich MG, Vinco G, Nadeshalingham G, White M, Mongeon F-P, Hillier E, Teixeira T, Flewitt JA, Eberle B, Fischer K (2019). Hyperventilation-induced heart rate response as a potential marker for cardiovascular disease. Sci. Rep..

[12] Dubé B-P, Agostoni P, Laveneziana P (2016). Exertional dyspnoea in chronic heart failure: The role of the lung and respiratory mechanical factors. Eur. Respir. Rev..

[13] Andreas S, Figulla HR (1994). Role of hyperventilation in the pathogenesis of central sleep apneas in patients with congestive heart failure. Am. J. Respir. Crit. Care Med..

[14] van Iterson EH, Johnson BD, Borlaug BA, Olson TP (2017). Physiological dead space and arterial carbon dioxide contributions to exercise ventilatory inefficiency in patients with reduced or preserved ejection fraction heart failure. Eur. J. Heart Fail..

[15] Torres-Torrelo H, Ortega-Sáenz P, Gao L, López-Barneo J (2021). Lactate sensing mechanisms in arterial chemoreceptor cells. Nat. Commun..

[16] Lorenzi-Filho G, Azevedo ER, Parker JD, Bradley TD (2002). Relationship of carbon dioxide tension in arterial blood to pulmonary wedge pressure in heart failure. Eur. Respir. J..

[17] Naughton M, Benard D, Tam A, Rutherford R, Bradley TD (1993). Role of hyperventilation in the pathogenesis of central sleep apneas in patients with congestive heart failure. Am. Rev. Respir. Dis..

[18] Lorenzi-Filho G, Genta PR, Figueiredo AC, Inoue D (2005). Cheyne–Stokes respiration in patients with congestive heart failure: Causes and consequences. Clinics.

[19] Rubio-Gracia J, Demissei BG, ter Maaten JM, Cleland JG, O’Connor CM, Metra M, Ponikowski P, Teerlink JR, Cotter G, Davison BA, Givertz MM, Bloomfield DM, Dittrich H, Damman K, Pérez-Calvo JI, Voors AA (2018). Prevalence, predictors and clinical outcome of residual congestion in acute decompensated heart failure. Int. J. Cardiol..

[20] Ambrosy AP, Cerbin LP, Armstrong PW, Butler J, Coles A, DeVore AD, Dunlap ME, Ezekowitz JA, Felker GM, Fudim M, Greene SJ, Hernandez AF, O’Connor CM, Schulte P, Starling RC, Teerlink JR, Voors AA, Mentz RJ (2017). Body weight change during and after hospitalization for acute heart failure: Patient characteristics, markers of congestion, and outcomes. JACC Heart Fail..

